# Ultrasound-guided omental biopsy: Review of 173 patients

**DOI:** 10.4103/0971-3026.73533

**Published:** 2010-11

**Authors:** Padmapriya Govindarajan, Shyamkumar N Keshava

**Affiliations:** Department of Radiology, Christian Medical College, Vellore, Tamil Nadu - 632 004, India

**Keywords:** Biopsy, greater omentum, ultrasound

## Abstract

**Background:**

Omental biopsy has conventionally been performed using a surgical approach. USG-guided omental biopsy is a safe and effective alternative. The purpose of this study was to assess the utility of USG guidance for biopsy of the greater omentum.

**Study design:**

Retrospective study.

**Materials and Methods:**

We retrospectively reviewed all omental biopsies performed under USG guidance from April 2006 to March 2010 in a tertiary care hospital.

**Results:**

One hundred and seventy-three patients were included. Out of these, 82 (47%) patients were diagnosed to have malignancies, 58 (34%) patients had granulomatous inflammation either suggestive of or consistent with tuberculosis, 29 (17%) patients were diagnosed to have inconclusive biopsy results, and 4 (2%) patients had an inadequate sample for histopathological examination. There were no major procedure-related complications.

**Conclusion:**

USG-guided biopsy of the omentum is a safe and effective procedure. A thickened omentum can serve as an easily accessible site for biopsy, especially in patients who have ascites of unknown etiology and in those with a history of previous malignancy.

## Introduction

The greater omentum is the largest of the peritoneal folds. Since a small amount of fluid is present in the peritoneal cavity, infections and malignancy easily spread to the omentum.[1] Ovarian and gastrointestinal malignancies are the most common neoplasms that seed the peritoneum.[2] The omentum can also be affected by other conditions, such as granulomatous inflammation, infections and hematoma. An omental mass in a patient with a known malignancy usually indicates metastasis; however, a biopsy is often necessary to confirm the diagnosis.[2]

An omental biopsy can be attempted once the omentum is thickened. Conventionally, omental biopsy has been performed using laparotomy or laparoscopy, which also involve additional costs of hospitalization and the risks of anesthesia. The omentum is easily visible on USG when it is thickened. Since the omentum is easily accessible and it can be easily differentiated from bowel on real-time USG, an omental biopsy can be readily performed under USG guidance. The purpose of this study was to evaluate the usefulness of USG guidance for the biopsy of a thickened omentum.

## Methods

We retrospectively reviewed patients who underwent USG-guided omental biopsies in our institution between April 2006 and March 2010. All patients who had a thickened omentum and no other accessible site for a biopsy were included in the study. The main exclusion criterion was the presence of uncorrectable bleeding parameters.

All the patients underwent a USG examination prior to the biopsy to assess the omental thickness and feasibility of the biopsy. The omentum was considered thickened if it measured more than 10 mm. Bleeding parameters including prothrombin time (PT), partial thromboplastin time (PTT), and platelet count were recorded for all patients. A platelet count above 80000/μl was considered acceptable for performing the procedure. Any PT value with an INR (international normalized ratio) less than 1.4 for patients, who were on oral anticoagulants, was considered acceptable as well. A PTT value of 23.8–37.4 s was considered acceptable for the procedure. All the three parameters were taken into account before the procedure. If any of the parameters was deranged, a hematology opinion was asked for and the biopsy was performed only if the hematologist gave a go-ahead or after the parameters were corrected. Informed consent was obtained from each patient prior to the procedure.

Sedation with pethidine and promethazine was administered half an hour prior to the procedure in order to reduce procedure-related pain and discomfort. The procedure was performed in the supine position. Using USG guidance, the omentum was assessed to identify the site of maximum thickening. The needle path was also assessed using color Doppler to ensure that there were no blood vessels in the expected needle path. The presence of ascites in these patients was not considered a contraindication. Ascitic fluid was not routinely tapped unless asked for by the referring doctor.

**Figure 1:**
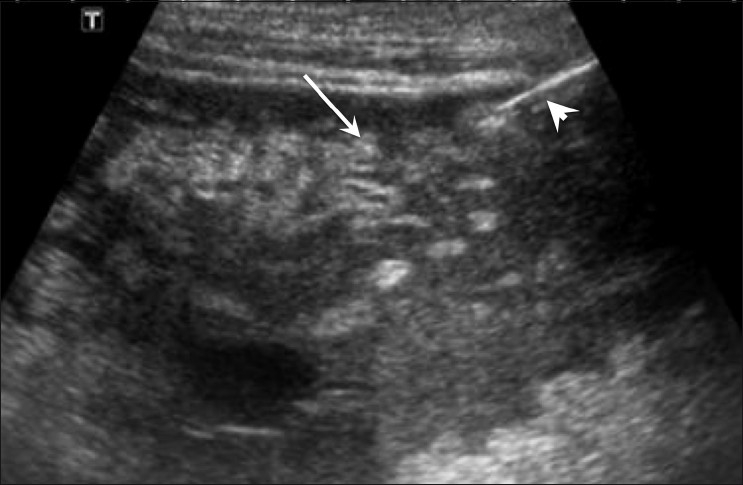
Sagittal USG image shows thickened omentum (arrow). The needle tip (arrowhead) is seen within

Bowel adjacent to the mass was identified by looking for peristalsis. The needle entry site was marked on the patient’s skin, and the surrounding area was cleansed with Betadine (povidone iodine). Local anesthetic (1–2% lidocaine hydrochloride) was injected with a 23G needle. The biopsies were performed using 3.5–7.0-MHz vector-phased array probes, with strict aseptic precautions. An 18G needle was advanced into the thickened omentum under real-time USG guidance using a free hand technique [Figure 1]. When the needle tip reached the omentum, the patient was asked to hold his/her breath in order to minimize injury to the omentum. A biopsy was performed using a BARD Magnum core biopsy needle and gun (BARD Magnum, Medical Device Technologies, USA). Two to four passes were made from the skin to the area of omental thickening. The biopsy specimen was sent for histopathological examination. Postprocedural USG was performed to look for any complication. The patients were monitored in the USG room for 30 min after which they were observed for 24 h in the daycare ward.

Retrospective data on the omental biopsies was collected and the histopathology reports were reviewed.

## Results

**Figure 2:**
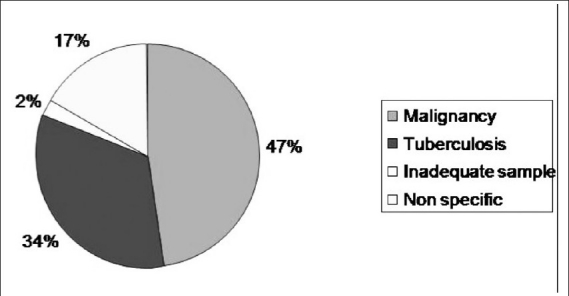
Pie-diagram shows the distribution of pathology of the omental biopsies

In this study we included 173 patients who had undergone omental biopsy. An adequate sample was obtained in 98% of the cases. Positive histopathological results were obtained in 140/173 (81%) patients. These included malignancy in 82/173 (47%) patients and granulomatous inflammation either suggestive of or consistent with tuberculosis in 58/173 (34%) patients. In 33/173 (19%) patients the biopsy was non-contributory. In 29/173 (17%) patients, the pathological reports were nonspecific and in 4/173 (2%) patients the sample was insufficient [Figure 2]. Granulomatous inflammation was only reported as either consistent with or suggestive of tuberculosis.

There were no major complications. One patient developed abdominal pain, which subsided with analgesics – there were no other minor complications.

## Discussion

Omental thickening is usually an indicator of an abdominal pathology such as malignancy or granulomatous inflammation. The omentum is involved when tumor cells seed the omentum via intraperitoneal dissemination, along with peritoneal reflections and ligaments and also hematogenously.[3] In tuberculosis, the omentum is involved by hematogenous spread from the lungs, by the lymphatics or direct spread.[3]

Although surgical biopsy is the gold standard, USG-guided biopsy is gaining widespread acceptance since it is quicker and less expensive. USG guidance has the advantage that it allows visualization of the needle during the procedure and, moreover, it is not associated with radiation hazards as with a CT-guided biopsy, though the results have been reported to be similar with both techniques.[2,4,5]

The literature regarding the use of USG for omental biopsy is limited.[4] Sistrom *et al*. in their series of 11 patients, who were monitored over a period of two years obtained positive results in nine patients.[6] Gottlieb *et al*. achieved a sensitivity of 93% and a specificity of 100%, using a 20G or 22G spinal needle for fine needle aspirates or an 18G core biopsy needle for biopsy, or both in 54 extra visceral masses in 52 patients.[7] They had nondiagnostic samples in 4% of patients and there was no procedure-related complications. Lisa *et al*. had 12 concordant diagnoses in 13 patients, where open surgical biopsy was performed on mesenteric masses, after USG-guided biopsy. Complications included mesenteric hematoma and abdominal wall cellulitis.[4] In our series, we obtained positive diagnostic results in 81% of the patients, although the specimen was considered adequate in 98%.

One limitation of our study is the non-inclusion of a gold standard for omental biopsy. This was not possible because the procedure of USG-guided biopsy is well established and well accepted in our institution. Since we did not follow-up patients beyond 24 hours, we may have missed delayed complications.

In conclusion, USG-guided biopsy of the omentum is a safe and effective method for the assessment of omental lesions visible on USG.
